# Advances in recombinant Bacillus Calmette–Guérin therapy for bladder cancer from 2015 to 2024: Innovations and challenges

**DOI:** 10.14440/bladder.2024.0072

**Published:** 2025-05-20

**Authors:** Hanzhao Zhu, Jiani Li, Xintong Zhou, Kaixia Mi

**Affiliations:** 1 Laboratory of Pathogen Microbiology and Immunology, Institute of Microbiology, Chinese Academy of Sciences, Beijing 100101, China; 2 Medical School, University of Chinese Academy of Sciences, Beijing 101408, China

**Keywords:** Bacillus Calmette–Guérin, Bladder cancer, Immune therapy, Recombinant Bacillus Calmette–Guérin

## Abstract

**Background:**

Non-muscle invasive bladder cancer is commonly treated by Bacillus Calmette–Guérin (BCG) therapy, but its efficacy is limited. Research into recombinant BCG (rBCG) aims to enhance its effectiveness and minimize side effects through genetic modifications.

**Objective:**

This review explored advancements in rBCG therapy (2015 – 2024), focusing on genetic modifications designed to enhance cytokine production, introduce bacterial effectors, and boost immune responses. It also discusses future research on alternatives such as *Mycobacterium smegmatis* for safety concerns and integrating rBCG with other therapies to improve patient outcomes.

**Conclusion:**

While rBCG therapies offer promising potential, overcoming translational challenges entails interdisciplinary endeavor to address the issues of safety, cost, and accessibility, and ultimately maximize their benefits for bladder cancer patients.

## 1. Introduction

Bladder cancer represents the 10^th^ most common malignancy across the globe and a leading cause of cancer-related deaths. It is categorized into non-muscle invasive bladder cancer (NMIBC) and muscle invasive bladder cancer (MIBC). NMIBC, which accounts for 70% of cases of bladder cancer, is commonly treated with Bacillus Calmette–Guérin (BCG) immunotherapy. However, the efficacy of BCG is limited, with 30 – 45% of patients failing to respond to treatment and 20% suffering from severe side effects. This reality highlights the urgent need to enhance the effectiveness of BCG therapy.^1^

**Figure 1:**
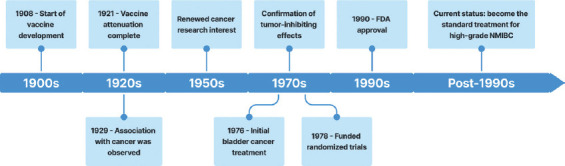
Timeline of Bacillus Calmette–Guérin vaccine development and its application in cancer therapy. 1908: Start of vaccine development. Albert Calmette and Camille Guérin isolated *Mycobacterium bovis* from infected cows’ udders at the Pasteur Institute in France, marking the inception of BCG vaccine development. 1921: Vaccine attenuation complete. After 231 passages over a period of 13 years, *M. bovis* was successfully attenuated, leading to the creation of the first stable BCG strain. 1929: Association with cancer was observed. Pearl observed that tuberculosis patients had lower cancer rates, which prompted the investigation into BCG’s potential as a cancer therapy. 1950s: Renewed cancer research interest. The development of animal models sparked renewed interest in BCG for cancer therapy, particularly its potential to inhibit tumor growth. 1970s: Confirmation of tumor-inhibiting effects. Burton Zbar confirmed BCG’s tumor-inhibiting effects in animal models, which played a pivotal role in advancing clinical research. 1976: Initial bladder cancer treatment. Alvaro Morales published groundbreaking research on using BCG to treat superficial bladder cancer, solidifying his key role in translating laboratory findings into clinical practice. 1978: Funded randomized trials. Morales’ advocacy led the National Cancer Institute to fund two randomized controlled trials, testing BCG’s effectiveness against superficial bladder tumors. 1990: FDA approval. The United States Food and Drug Administration approved intravesical BCG therapy, supported by trials involving over 2,500 patients, making it the standard treatment for high-grade NMIBC. Post-1990: Current status. Over the past 30 years, BCG therapy has become the standard treatment for high-grade NMIBC, and has been extensively used and studied in cancer therapy. Abbreviations: BCG: Bacillus Calmette–Guérin; FDA: Food and Drug Administration; NMIBC: Non-muscle invasive bladder cancer.

BCG, developed over a century ago, was first recognized to possess antitumor effects in 1929. In 1976, it received the Food and Drug Administration’s approval for the treatment of bladder cancer (Figure 1).^1^ While BCG remains the standard therapy for NMIBC, its underlying mechanisms have not been fully understood. In recent years, efforts have been directed at enhancing BCG therapy using novel formulations and recombinant BCG (rBCG), the latter involving genetic modifications to improve efficacy and minimize side effects. This review highlights recent advancements in rBCG therapy from 2015 to 2024, focusing on cytokine production and bacteriological mechanisms aimed at improving the safety and efficacy of BCG therapy for bladder cancer.

## 2. Effector mechanisms and innovations in BCG therapy for bladder cancer

Effector mechanisms in BCG therapy enhance the immune response against bladder cancer by activating immune cells, such as T cells and macrophages, which play an important role in tumor cell clearance. Key cytokines – including interleukin (IL)-2, interferon (IFN)-α, IFN-γ, IL-12, IL-15, and tumor necrosis factor (TNF)-α – are vital for treatment efficacy. The following section discusses recent innovations in modifying BCG strains to enhance cytokine production and bacterial effector mechanisms in combating bladder cancer.

### 2.1. Modifying BCG to enhance cytokine production

Cytokines significantly contribute to BCG therapy by directly participating in antitumor responses and enhancing the immune system’s attack on tumors. Modifying BCG to enhance cytokine production has led to promising advances in bladder cancer immunotherapy.

#### 2.1.1. BCG-IL-15

A pioneering study by Takeuchi *et al*.^2^ introduced a novel rBCG, known as BCG-IL-15, which secretes an IL-15 fusion protein. The study demonstrated that BCG-IL-15 significantly prolonged survival in a mouse model of bladder cancer compared to traditional BCG therapy. In addition, BCG-IL-15 significantly increased the infiltration of neutrophils and γδ T cells, two key players in the production of IL-17. It also enhanced the expression of chemokines, such as macrophage inflammatory protein-2 and macrophage inflammatory protein-1α, involved in the recruitment of neutrophils to the tumor site. These findings suggest that BCG-IL-15 could offer a promising therapeutic approach for NMIBC by enhancing the immune response through the secretion of IL-15.

#### 2.1.2. BCG-human IFN-α-2b (hIFNα-2b)

Sun *et al*.^3^ developed an rBCG engineered to secrete hIFNα-2b, referred to as rBCG-hIFNα-2b. Preclinical studies demonstrated that this modified version achieved improved therapeutic efficacy when compared to traditional counterparts. *In vitro* studies showed that rBCG-hIFNα-2b inhibited the proliferation of the MB49 bladder cancer cell line and induced apoptosis at higher rates compared to conventional BCG. In a mouse model, rBCG-hIFNα-2b not only prolonged survival and reduced bladder mass but also up-regulated the expression of apoptosis-related Fas protein. Furthermore, rBCG-hIFNα-2b treatment elevated the levels of inflammatory cytokines, such as TNF-α and IL-12, in peripheral blood. It also increased the number of polymorphonuclear leukocytes, monocytes, and T lymphocytes, while enhancing the CD4^+^/CD8^+^ T cell ratio – a critical indicator of an activated immune response. These results suggest that rBCG-hIFNα-2b could be a superior therapeutic option for NMIBC. Further studies are warranted to confirm these results and look into the underlying mechanisms.

### 2.2. Modifying BCG to produce bacterial effectors

rBCG strains that express bacterial effectors have emerged as an innovative strategy to enhance immune responses. These engineered BCG strains are designed to express specific antigens, thereby triggering both specific and non-specific immune responses, which eventually lead to improved efficacy of cancer therapy.

#### 2.2.1. VPM1002 and its modified form, VPM1002BC

The rBCG strain VPM1002 and its modified variant, VPM1002BC, are utilized for the treatment of NMIBC.

*2.2.1.1. Gene modification and mechanisms*

rBCG strain VPM1002 presents an innovative gene modification strategy for bladder cancer treatment, where the urease C gene is deleted and replaced with the listeriolysin gene.^4^ VPM1002 was further modified into VPM1002BC, a strain specifically designed for bladder cancer therapy. This modification enhances the vaccine’s ability to optimally present antigens to the immune system, potentially stimulating strong cancer-specific cytotoxic T-cell responses. As a result, VPM1002BC shows promising potential in improving the outcomes of bladder cancer patients.

*2.2.1.2. Preclinical and clinical developments*

Preclinical studies have demonstrated that VPM1002 offers improved safety and efficacy compared to traditional BCG therapy. Notably, VPM1002 holds promising potential in minimizing adverse effects while maintaining or even enhancing therapeutic effectiveness. In clinical trials, VPM1002 represents a significant advancement in the development of rBCG strains for bladder cancer treatment, highlighting the potential of rBCG to improve patient outcomes.^4^

*2.2.1.3. Phase I/II trial (SAKK 06/14)*

The phase I/II SAKK 06/14 trial^5^ is a multicenter, open-label, single-arm, dose-escalation study (NCT02371447) that evaluated the safety and efficacy of VPM1002BC in NMIBC patients who had not responded to conventional BCG therapy. The trial employed a 3+3 dose-escalation design, with weekly intravesical instillations of VPM1002BC to six patients for 6 weeks, followed by maintenance therapy over the following year. The trial results demonstrated that VPM1002BC was well-tolerated by NMIBC patients, with no dose-limiting toxicity or serious adverse events observed. A few grade 2 adverse events occurred, including asymptomatic urinary tract infections and prostatitis. Preliminary immunological data suggested a predominantly T helper type 1 immune response, which may indicate the potential effectiveness of VPM1002BC. These findings support the need for further evaluation of VPM1002BC in larger, randomized trials to assess its safety and efficacy in the treatment of NMIBC.

*2.2.1.4. Phase II trial (SAKK 06/19)*

The phase II SAKK 06/19 trial^6^ is a single-arm, multicenter clinical study evaluating an integrated regimen for MIBC. This regimen combines VPM1002BC with systemic therapies, including chemotherapy and immunotherapy, and is followed by surgery. Patients in the trial initially received treatment with VPM1002BC, followed by four cycles of cisplatin and gemcitabine chemotherapy, and four cycles of atezolizumab immunotherapy, each administered every 3 weeks. The patients then underwent radical cystectomy and pelvic lymph node dissection, with atezolizumab given as maintenance therapy for 13 cycles. Preliminary safety data from the first 12 patients suggest that this integrated treatment strategy is safe and holds promising potential for improving outcomes of MIBC patients, particularly those who had not responded well to conventional therapies. Ongoing studies aim to further validate these findings.

### 2.3. Other rBCG strains

#### 2.3.1. rBCG strains expressing the detoxified S1 subunit of the pertussis toxin (PT)

A study by Rodriguez *et al*.^7^ developed a rBCG strain modified with the detoxifying S1 subunit of PT, known as rBCG-S1PT. *In vitro* studies demonstrated that rBCG-S1PT enhanced the production of cytokines, such as IL-6, IL-8, and IL-10, while maintaining comparable levels of other cytokines compared to wild-type BCG. Furthermore, rBCG-S1PT improved activation markers on CD4^+^ T cells and exerted greater cytotoxicity against MB49 bladder cancer cells, suggesting it could potentially be an enhanced immunotherapy for bladder cancer.

In another study,^8^ several rBCG strains expressing S1PT were developed using different approaches, including rBCG-S1PT (extrachromosomal vectors), rBCG-S1i (integrating vectors), and rBCG-S1+S1i (combined strain). These strains exhibited, to various extents, localization and expression of S1PT. The extrachromosomal vectors targeted the cell wall-associated fractions, while integrating vectors are directed at intracellular locations. Mice vaccinated with rBCG-S1i showed significantly higher levels of IFN-γ compared to those treated with BCG or rBCG-S1PT. Moreover, the rBCG-S1+S1i group showed elevated levels of multifunctional CD4^+^ T cells expressing IFN-γ and TNF-α, demonstrating enhanced immunogenicity. These findings further support the potential of using rBCG-S1PT for the immunotherapy of bladder cancer.

In addition, rBCG-S1PT shows promising potential in enhancing innate immune memory.^9^ In a murine model, macrophages pretreated with rBCG-S1PT produced higher levels of IL-6, TNF-α, and IL-10 compared to those treated with wild-type BCG, thereby enhancing their responses to pathogens. Further *in vivo* testing demonstrated the protective effect of rBCG-S1PT against *Candida albicans*, with immunized mice showing increased IFN-γ and reduced *C. albicans* colony-forming units in their kidneys. These findings suggest rBCG-S1PT could potentially strengthen non-specific immune protection.

While these results are promising, further studies are warranted to confirm the efficacy and safety of rBCG-S1PT in humans. Ongoing research with various pathogen models, along with additional clinical trials, is essential to the validation of these findings and assessment of the broader clinical potential of rBCG-S1PT in immunotherapy.

#### 2.3.2. rBCG strains expressing Streptococcal inhibitor of complement and D-alanyl carrier protein ligase

The inefficacy of traditional BCG treatment in bladder cancer may be attributed to the presence of antimicrobial peptides (AMPs) on the surface of bladder cancer cells, which can inhibit BCG activity. To address this challenge, genetic modifications have been utilized to enhance BCG’s resistance to the cytotoxic effects of AMPs. Cho *et al*.^10^ developed two rBCG strains – rBCG-*sic* and rBCG-*dltA* – by incorporating genes for the *Streptococcal* inhibitor of complement (*sic*) and D-alanyl carrier protein ligase (*dltA*), respectively. These modifications were introduced into BCG through electroporation to improve its resistance to human AMPs.

*In vitro* evaluations using 5637 and T24 bladder cancer cell lines demonstrated that the rBCG strains not only achieved better survival rates in the presence of AMPs but also yielded significantly enhanced growth inhibitory effects. In addition, the rBCG strains promoted migration of human monocytic leukemia (THP-1) cells and showed increased internalization into bladder cancer cells following infection. Furthermore, both rBCG-*dltA* and rBCG-*sic* induced the secretion of antitumor cytokines, including IL-6, IL-12, TNF-α, and IFN-γ, highlighting their potential as improved immunotherapeutic options for bladder cancer.

Several studies^11^,^12^ evaluated the effectiveness of rBCG-*dltA* and rBCG-*sic* using high-throughput, three-dimensional bioprinted bladder cancer-on-a-chip models, as well as *in vivo* mouse models. The results demonstrated that rBCG-*dltA* significantly reduced the proliferation of T24 and 253J bladder cells while enhancing THP-1 cell migration and increasing the levels of TNF-α and IL-6. Similarly, rBCG-*sic* reduced the T24 cell viability, which was positively correlated with THP-1 cell migration and increased IL-6 levels. Both strains exhibited improved cytotoxicity compared to traditional BCG, further highlighting their enhanced anticancer properties in mouse models. These improvements are ascribed to the genetic modifications that confer AMP-induced resistance to the rBCG strains.

These findings contributed to the advancement of bladder cancer treatment strategies by demonstrating the potential of genetically-modified rBCG strains to enhance antitumor immune responses. The findings suggest that further research into their long-term efficacy and safety is needed. Moreover, combining rBCG treatments with other therapeutic approaches could potentially enhance the effectiveness of bladder cancer therapies and eventually improve patient outcomes. These promising results indicate that rBCG-based therapies could attain significant improvements in bladder cancer management.

## 3. Improving rBCG strains with additional immunity-boosting methods

### 3.1. rBCG-diadenylate cyclase

To further enhance the immunogenic potential of rBCG strains, Singh *et al*.^13^ engineered rBCG strains by incorporating the diadenylate cyclase (*DisA*) gene to stimulate STING-dependent immune responses. The overexpressing (OE) rBCG-*DisA* (rBCG-*DisA*-OE) strains were evaluated against wild-type BCG using various bladder cancer cell lines and mouse models. *In vivo* studies in N-methyl-N-nitrosourea-treated rat and MB49 mouse bladder cancer models demonstrated that rBCG-*DisA*-OE strains induced stronger IFN-I responses and elevated levels of pro-inflammatory cytokines such as TNF-α, IL-6, and IL-1β. These increased cytokines led to reduced tumor growth and enhanced immune activation.

In preclinical studies^14^ using rat and mouse bladder cancer models, rBCG-*DisA*-OE therapy exhibited significant tumor reduction and enhanced immune response. In rats, the therapy reduced tumor involvement and activated IFN-I signaling. In mice, the therapy not only further diminished tumor size but also raised IFN-γ levels and promoted CD4^+^ T cell infiltration into the tumor microenvironment. What is more, rBCG-*DisA*-OE caused epigenetic changes, including the modification of histone H3K4Me3, as observed *in vitro*. These alterations were associated with enhanced metabolic and immune responses of macrophages, suggesting that H3K4Me3 modifications could also shape the epigenetic landscape of immune cells, contributing to its anticancer effects.

Further research on rBCG therapies, particularly in a mouse model of MB49 bladder cancer, investigated the combination of programmed death ligand 1 checkpoint inhibitors with either wild-type BCG or rBCG overexpressing STING agonists.^15^ This combination therapy enhanced tumor suppression by promoting the recruitment of effector CD8^+^ T cells to the tumor site, thereby improving therapeutic efficacy.

While rBCG vaccines, with their ability to activate the STING pathway, showed promising antitumor effects against NMIBC in animal models, clinical trials in humans are still lacking. The lack of human clinical data means that these findings have yet to be confirmed in clinical practice. Therefore, further investigation is required to evaluate the potential of rBCG-based therapies and STING activation in the clinical treatment of bladder cancer.

### 3.2. rBCG strains overexpressing FimH on the surface

In addition to genetic modifications aimed at enhancing immune responses, research has also tried to improve the ability of BCG to specifically target bladder cancer cells. Maalouf *et al*.^16^ discovered that *Pseudomonas aeruginosa* expressing the mannose-sensitive hemagglutinin-type hairs could effectively target bladder cancer in mice by binding to the higher levels of mannose present on the surface of cancerous bladder cells. This concept was adapted for BCG, leading to the creation of rBCG-S.FimH, a strain that overexpresses the type 1 adhesin FimH.

In mouse models of NMIBC, Zhang *et al*.^17^ demonstrated that rBCG-S.FimH triggered a stronger Th1-type immune response, which is crucial for the effective elimination of cancer cells. In addition, rBCG-S.FimH activated dendritic cells, further enhancing the immune response through the Toll-like receptor 4-dependent pathway. Other studies have also demonstrated that rBCG-S.FimH possessed more potent antitumor activity *in vitro* compared to traditional BCG, suggesting that mannose-targeted therapies could significantly enhance treatment efficacy for bladder cancer.

These findings highlight the potential of mannose-targeted strategies being adopted in bladder cancer treatment and the promising therapeutic applications of rBCG-S.FimH. Future refining of these strategies could improve their clinical utility, particularly for NMIBC patients who are resistant to conventional BCG therapies.

## 4. Discussion

Recent advancements in rBCG therapy for bladder cancer showed that they could overcome the limitations of traditional BCG therapy, particularly by enhancing immunological efficacy and antitumor responses. Studies on BCG strains, such as VPM1002, are progressing to phase III trials, highlighting the potential of innovative therapeutic approaches. However, further investigations are needed to assess their clinical viability, safety, and long-term effects.

Despite the promising benefits of rBCG therapy, several limitations remain, particularly those concerning its safety and toxicity, especially for immunocompromised populations, such as human immunodeficiency virus (HIV)-positive patients. In these individuals, the use of traditional BCG is often restricted due to potential risks. As an alternative, *Mycobacterium smegmatis* has been explored as a treatment option for HIV-positive patients. This strain possesses immunity-stimulating properties without the risks associated with live BCG, rendering it a safer choice for immunocompromised individuals.^18^,^19^

Moreover, advancements in recombinant mycobacteria designed to enhance inflammatory responses and macrophage activity may be conducive to the development of safer and more effective vaccine alternatives.^20^ These developments offer a transformative opportunity for bladder cancer treatment, potentially addressing current limitations and improving patient outcomes. However, to accomplish widespread clinical application, significant challenges remain, including regulatory approval, economic feasibility, and scalability.

Understanding the cost-effectiveness and accessibility of rBCG therapy across diverse healthcare settings is essential for its successful implementation. Addressing these challenges will require strategic planning to integrate rBCG into clinical practice in a manner that is both financially and logistically sustainable.

## 5. Conclusion

rBCG therapies hold promising potential for pushing forward bladder cancer treatment, but addressing translational challenges is crucial. Advancing BCG research will require interdisciplinary collaboration among immunologists, clinicians, and regulatory bodies to address issues related to safety, cost, and accessibility, to maximally benefit bladder cancer patients.

## Data Availability

Not applicable.
